# Phthalocyanine-Based Molecularly Imprinted Polymers as Nucleoside Receptors

**DOI:** 10.1155/2008/281843

**Published:** 2007-11-22

**Authors:** Luigia Longo, Giuseppe Vasapollo

**Affiliations:** Dipartimento di Ingegneria dell'Innovazione, Università del Salento, 73100 Lecce, Italy

## Abstract

A molecularly imprinted polymer (MIP) for tri-O-acetyladenosine (TOAA), PPM(TOAA), was prepared by the combined use of methacrylic acid (MAA) and Zn(II)tetra(4′-methacryloxyphenoxy) phthalocyanine as functional monomers. This MIP exhibited a higher binding ability for TOAA compared to the MIP prepared using only MAA, PM(TOAA), in batch rebinding tests. Scatchard analysis gave a higher association constant of PPM(TOAA) for TOAA $\left(2.96\times 10^{4}{\text{ M}}^{-1}\right)$ than that of PM(TOAA) $\left(1.48\times 10^{4}{\text{ M}}^{-\text{1}}\right)$. The MIP prepared using only the zinc-phthalocyanine, PP(TOAA), did not show any binding capacity for TOAA. This means that the phthalocyanine in the MIP contributes to higher affinities, although it barely interacts with TOAA. Since selectivity for this kind of MIPs is more important than binding affinity, the binding of TOAA and a structurally related compound, tri-O-acetyluridine (TOAU), on the polymers was investigated. Both PPM(TOAA) and PM(TOAA) exhibited binding affinities for TOAA while they did not show any binding capacity for TOAU.

## 1. INTRODUCTION

Molecularly imprinted polymers (MIPs) have received much attention recently as new materials capable of molecular recognition [1]. They have been extensively utilized in solid-phase extraction [2–5], chromatography [6–8], sensing [9, 10], and catalysis [11–13]. During molecular imprinting, crosslinked polymers are formed by free-radical copolymerization of functional monomers with an excess of crosslinker around an analyte that acts as a template. After polymerization, the template is removed leaving in this way selective binding sites in the polymer network that are complementary in form and functionality to the analyte molecules [1].

The main advantages of MIPs, over conventional polymers used as separation material, are the high selectivity and affinity for the target analytes used in the imprinting procedure. Furthermore, MIPs are characterized by superior mechanical and thermal stability, as well as better inertness towards acids, bases, metal ions, and organic solvents compared to enzymes. In addition, imprinting polymerization is a very inexpensive procedure for the development of artificial receptors. In the majority of the cases, the price of an MIP depends entirely on the price of the template used. Moreover, if the template is expensive, it is possible to recover the template and use it again. Alternatively, inexpensive template analogues can be used for the preparation of MIPs [1].

In most studies, methacrylic acid (MAA) was used as functional monomer to synthesize MIPs making the polymer preparation a simple and facile process [14–16]. MAA can form hydrogen bonds with the template molecule in porogen prior to polymerization. A more deliberate approach using synthetically designed functional monomers could enable a better control in the formation of high-affinity binding sites for each corresponding template minimizing, at the same time, the inherent nonspecific binding properties common in noncovalent imprinted polymers.

**Scheme 1:**
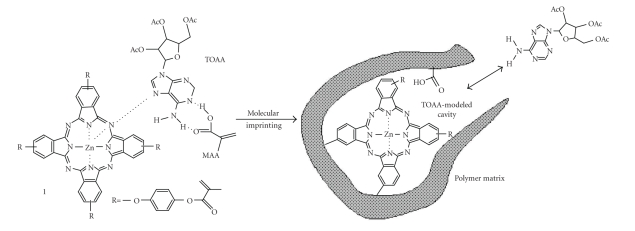
Schematic representation of the molecular imprinting of TOAA using both 1 and MAA as functional monomers.

In this paper, we report for the first time the preparation of MIPs as nucleoside receptors using both methacrylic acid and a zinc-phthalocyanine peripherally substituted with methacrylic groups (Compound 1, Scheme 1) [17] as functional monomers. The receptor site is a three-dimensional cavity around the phthalocyanine plane in crosslinked polymers to which the analyte molecule could be specifically bound via coordination through the metal of the phthalocyanine and hydrogen bonding/electrostatic interaction with MAA and the modifiers linked to the phthalocyanine (Scheme 1). Such cooperative interaction approach in MIP formation, already proposed for porphyrin derivatives [18–21], was applied here for the first time to phthalocyanine compounds.

An organic soluble nucleoside derivative, tri-O-acetyladenosine (TOAA), was utilized in this work as template in the preparation of phthalocyanine-based MIPs. The development of synthetic receptors that recognize nucleotide bases and their derivatives is an important area in chemistry today. The literature provides many examples of artificial receptors for each of the common nucleoside bases [15,16,22–25]. Recently, these receptors have provided insights into DNA-DNA and protein-DNA interactions, and applications are envisioned in the fields of biosensors, drug therapy, separation science, and genetic engineering. TOAA was selected as model template in a previous work [17] where the binding affinity and selectivity of the zinc-phthalocyanine 1 towards different nucleosides were evaluated by UV-vis titration experiments. Binding experiments showed that zinc-phthalocyanine 1 bounds TOAA most strongly, giving a binding constant $K_{a}$ of $1.35\times 10^{4}$, 500 times that of 1 to tri-O-acetyluridine (TOAU), showing in this way a high selectivity [17]. The presence of the 2-aminopyridine moiety in the structure of TOAA could be probably responsible for the good binding characteristics of this nucleoside derivative with compounds functionalized with methacrylic groups [15].

An imprinted polymer receptor for TOAA, namely, PPM(TOAA), was prepared using both 1 and MAA as functional monomers. Imprinted polymers were also prepared using either MAA or 1, called PM(TOAA) and PP(TOAA), respectively, and used as references. Corresponding unimprinted blank polymers, PPM(BL), PM(BL), and PP(BL), were prepared using the same monomers in the absence of TOAA. Batch rebinding studies were conducted by UV-vis spectroscopy and the binding characteristics of the MIPs were examined by Scatchard analysis. In order to verify the selectivity of the MIPs, the binding of TOAA and its structurally related compound, TOAU, on the all prepared polymers was investigated.

## 2. EXPERIMENTAL SECTION

### 2.1. Materials and methods

Ethylene glycol dimethacrylate (EGDMA), 2′,3′,5′-tri-O-acetyladenosine (TOAA), 2′,3′,5′-tri-O-acetyluridine (TOAU), and acetic acid were purchased from Sigma-Aldrich (Steinheim, Germany). *α*-*α*’-Azoisobutyronitrile (AIBN) and methacrylic acid (MAA) were supplied from Fluka (Steinheim, Germany). Analytical grade dichloromethane and methanol were purchased from J. T. Baker (Deventer, Holland). Zn(II) tetra(4′-methacryloyloxyphenoxy)phthalocyanine (1) was prepared on the basis of a published method [17]. UV-Vis spectra were obtained with a Cary 100 Scan UV-vis spectrophotometer.

### 2.2. Polymer preparation

The preparation of PPM(TOAA) was carried out as follows: to a solution of TOAA (0,0468 mmol, as template) in dichloromethane (262 *μ*L) were added phthalocyanine 1 (0.0117 mmol, as the first functional monomer) and MAA (0.1758 mmol, as the second functional monomer) in a glass tube. After adding of EGDMA (0.9360 mmol, as cross-linker) and AIBN (0.0112 mmol, as initiator), the mixture was sonicated for 5 minutes flushing with nitrogen gas and then polymerized by heating at 60°C for 16 hours. The resultant polymer was crushed and sieved. The template molecules were removed by washing the polymer first with methanol/acetic acid (7/3 v/v) and then with methanol until no template molecules were detected from the recovered solutions with a UV-vis spectrophotometer. Drying under vacuum afforded particles which were used for rebinding studies. PM(TOAA) and PP(TOAA) were identically prepared using either MAA (0.1758 mmol) or 1 (0.0117 mmol) as functional monomer, respectively. The corresponding blank polymers, PPM(BL), PM(BL), and PP(BL), were prepared in the same manner in the absence of TOAA.

### 2.3. Batch rebinding experiments and Scatchard analysis

The polymer (20 mg) was added to a dichloromethane solution (3.5 mL) of TOAA of known concentrations ($2.0–8.0\times 10^{5}$ M) in vials. The resulting suspension was shaken for 16 hours at room temperature, then the polymer was rapidly removed by filtration and the resulting solution was analyzed by UV-vis spectrophotometer at 258 nm. The amount of TOAA bound to the polymer, *B*, was calculated by subtraction of the concentration of free TOAA, [TOAA], from the initial TOAA concentration. [TOAA] was determined as an average value of three measurements. Scatchard analysis was provided by the Scatchard equation, *B*/[TOAA] = ($B_{\max}$−*B*) $K_{a}$, where $K_{a}$ is the association constant and $B_{\max}$ is the apparent maximum number of binding sites. Therefore, $K_{a}$ and $B_{\max}$ of the polymer were determined from the slope and the intercept, respectively, by plotting of *B*/[TOAA] versus *B*. Batch rebinding experiments and Scatchard analysis were performed in a similar manner for PPM(TOAA), PM(TOAA), PP(TOAA), and the corresponding blank polymers. The rebinding tests were also carried out incubating the polymers with TOAU in order to verify their binding selectivity.

## 3. RESULTS AND DISCUSSION

**Figure 1:**
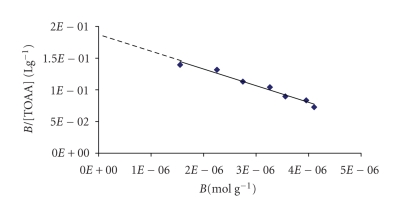
Scatchard plot for PPM(TOAA).

TOAA-imprinted and unimprinted polymers were obtained by the above method. The binding behavior of the prepared MIPs was evaluated by batch rebinding tests and the binding data were processed with Scatchard equation in order to estimate the binding properties of the polymers. Figure 1 shows the Scatchard plot for PPM(TOAA). As can be seen, it is a single straight line, which indicates that there exists one kind of binding sites populated in the MIP. The Scatchard plot is linear also in the case of PM(TOAA). Similar Scatchard plots were obtained in a study of Yan et al. [26] with malachite green-imprinted polymers. This fact is very interesting since a nonlinear profile was commonly observed in the Scatchard assessment of MIPs indicating the presence of binding sites that exhibit various affinities to the ligand [18, 20, 21]. Table 1 shows the values of $K_{a}$ and $B_{\max}$ for PPM(TOAA) and PM(TOAA). As shown, the MIP prepared with both functional monomers 1 and MAA, PPM(TOAA), exhibited higher binding affinities for TOAA compared to PM(TOAA), prepared solely with MAA. PP(TOAA), prepared only with 1, did not show any binding capacity for TOAA. This means that phthalocyanine 1 contributes to higher binding affinities, although 1 itself barely interacts with TOAA. The effects of the use of both functional monomers strongly suggest that the imprint that allows the simultaneous multipoint interactions of the template with the carboxylic residues of MAA and the Zn(II) ion of 1 shows a higher binding ability for TOAA than that allowing only the individual template/functional monomer interactions. The corresponding blank polymers, PPM(BL), PM(BL), and PP(BL), showed no binding affinities for TOAA confirming that the selectivity was due to the imprinting of the polymer matrix and not to the intrinsic affinity of the template to the functional monomers alone.

**Figure 2:**
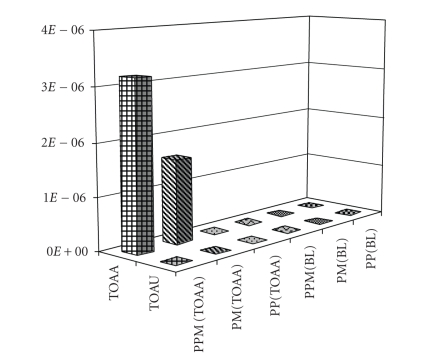
Binding selectivity test of TOAA and TOAU on the MIPs.

As for the selectivity (Figure 2), both PPM(TOAA) and PM(TOAA) exhibited binding affinities for TOAA while they did not show any binding capacity for TOAU. As expected, also PP(TOAA) did not show any binding capacity for TOAU. The corresponding blank polymers showed no binding affinity for TOAU too. If we examine the chemical structure of TOAA and TOAU, it is clear that TOAA has more chances than TOAU to form hydrogen bonds with the MIPs because of more functional groups that could form hydrogen bonds with the polymers. Consequently, the polymers PPM(TOAA) and PM(TOAA) showed a good binding affinity for TOAA and no binding capacity for TOAU. This is not surprising since the inability of carboxylic acid receptors to bind uracil derivatives appears to be rather general as evidenced in the literature [15, 27, 28].

The higher binding affinity of PPM(TOAA) for TOAA in comparison with PM(TOAA) could be explained considering the possibility of coordination of the Zn(II) ion of 1 with the nitrogen atoms of the 2-amonopyridine moiety of TOAA. Similar multipoint interactions have already been proposed by Takeuchi et al. [18] in a study on the preparation of MIPs for cinchonidine by the combined use of MAA and a vinyl-substituted zinc(II) porphyrin as functional monomers. The coordination of the Zn(II) ion with the nitrogen atoms of TOAA could be explained considering that the zinc(II) ion is the acid with moderate hardness and the nitrogen atom of the 2-aminopyridine moiety is the base with moderate softness [18]. The absence of this kind of nitrogen atom in the chemical structure of TOAU could explain the inability of PPM(TOAA) to bind TOAU. On the other hand, the inability of PP(TOAA) to bind TOAA suggests that the coordination of the Zn(II) ion of the phthalocyanine with the nucleoside is not sufficient alone to assure the recognition and subsequent complementary binding between the receptor and the nucleoside molecule, confirming in this way that the analyte molecule is specifically bound to the polymer through multipoint interactions.

## 4. CONCLUSION

A highly specific and selective TOAA-imprinted polymer was prepared by the combination of MAA and a zinc-phthalocyanine substituted with methacrylic groups as functional monomers. The effects of the simultaneous use of the two functional monomers suggest the effective cooperation of the phthalocyanine-based and carboxylic residues rather than independent operation for retaining of TOAA.

Considering these promising results for easily constructed and highly selective MIPs for nucleosides, new investigations are now being directed towards the development of MIP-based sensors arrays for nucleoside discriminations.

## Figures and Tables

**Table 1:** Association constant ($K_{a}$) and maximum number of binding sites ($B_{\max}$) for PPM(TOAA) and PM(TOAA).

Polymer	$K_{a}$(M^-1^)	$B_{\max}$ (*μ*Mg^-1^)
PPM(TOAA)	(2.96 ± 0.5) × 10^4^	6.53
PM(TOAA)	(1.48 ± 0.6) × 10^4^	1.66
